# Superposition mechanism as a neural basis for understanding others

**DOI:** 10.1038/s41598-022-06717-3

**Published:** 2022-02-21

**Authors:** Wataru Noguchi, Hiroyuki Iizuka, Masahito Yamamoto, Shigeru Taguchi

**Affiliations:** 1 Faculty of Information Science and Technology, Hokkaido University, Sapporo, 060-0814 Japan; 2 Center for Human Nature, Artificial Intelligence, and Neuroscience, Hokkaido University, Sapporo, 060-0812 Japan; 3 Faculty of Humanities and Human Sciences, Hokkaido University, Sapporo, 060-0810 Japan

**Keywords:** Learning algorithms, Network models

## Abstract

Social cognition has received much attention in fields such as neuroscience, psychology, cognitive science, and philosophy. Theory-theory (TT) and simulation theory (ST) provide the dominant theoretical frameworks for research on social cognition. However, neither theory addresses the matter of how the concepts of “self” and “other” are acquired through the development of human and nonhuman agents. Here, we show that the internal representations of “self” and “other” can be developed in an artificial agent only through the simple predictive learning achieved by deep neural networks with the *superposition mechanism* we herein propose. That is, social cognition can be achieved without a pre-given (or innate) framework of self and other; this is not assumed (or is at least unclear) in TT and ST. We demonstrate that the agent with the proposed model can acquire basic abilities of social cognition such as shared spatial representations of self and other, perspective-taking, and mirror-neuron-like activities of the agent’s neural network. The result indicates that the superposition mechanism we propose is a necessary condition for the development of the concepts of “self” and “other” and, hence, for the development of social cognition in general.

## Introduction

The problem of other minds has puzzled philosophers and scientists for centuries. Since the twentieth century, studies on this problem have been based on two dominant theories: theory-theory (TT)^1–3^ and simulation theory (ST)^4–6^. Theorists of TT argue that understanding or “mindreading” of others is achieved through naïve-psychological (or “folk-psychological”) conceptual schemes that agents use to understand other agents’ unobservable mental states. In contrast, theorists of ST claim that in order for agents to understand the mental states of others, they need a kind of mental simulation in which the agents use their own mental states to infer the corresponding mental states of others. Based on these ideas, neuroscientists have attempted to uncover the brain processes related to other minds from the perspectives of TT and ST^7–10^. However, one important point seems to be missing from these theories. Both presuppose that “self” and “other” are pre-given as two different frames. Based on this presupposition, they attempt to clarify how the self can make inferences about other minds using only the resources available within the self. However, do we have pre-given frames of self and others from the beginning of our experience as newborns? It is even possible in adult social cognition that information about self and others is not always processed separately based on the separate frames of “self” and “other” given in advance. For example, de Bézenac et al. argue that ambiguity in self-other processing plays an important role in the adaptive, flexible, and healthy sense of self in children and adults^11^.

There are some researchers who challenge the standard view of TT and ST and argue against their individualist and mentalizing explanations of social cognition. These researchers are proponents of interaction theory (IT)^12–16^, which claims that interactions between agents play a fundamental role in human development and sociality. They criticize the view that human agents unilaterally observe other agents and make inferences about others’ mental states using their own internal resources, such as theories of mind and simulation mechanisms. We believe that this is an important theoretical advance. However, interaction theorists do not (at least explicitly) relativize the assumption that there are pre-given frames that are recognized as self and other. They stress that mutual interactions play a constitutive role in social cognition from the beginning of human life but do not seem to be attempting to demolish the pre-existing concepts of “self” and “other.” We question this remaining assumption. It is more natural to assume that the contrasting concepts of “self” and “other” can be obtained only in a process of learning. In other words, we can assume that a human agent does not have the concepts of self and other from the beginning of its existence; rather, they are acquired through the process of learning by gradually being contrasted against and related to each other.

**Figure 1:**
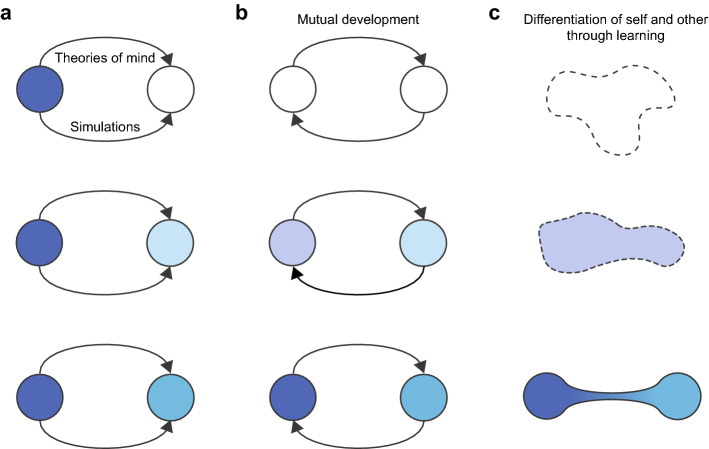
Different views of development of the self and the other. (**a**) The self pre-exists, and representation of the other is acquired by using theories of mind or simulations. (**b**) Two different frames of the self and other are pre-given, and their contents are developed through mutual interaction. (**c**) The self and the other are not pre-given frames but are differentiated through learning. In this process, the contents of their minds are synchronized and differentiated at the same time.

In summary, the standard views (TT and ST) assume that the self pre-exists and acquires representations of others step by step using theories of mind or simulations. This can be illustrated as follows: a pre-existing frame (a self) is filled with contents from the beginning, and the self uses its own resources to fill the content of another empty frame (the other) (Fig. 1a). Some researchers, namely, interaction theorists, oppose this view and claim that there are mutual relationships between the self and others right from the outset and that these frames (self and other) are simultaneously filled with contents through interaction in the development process (Fig. 1b). We do not enter into the debates between TT, ST, and IT in this paper. However, we would like to point out that none of these theories explicitly questions the pre-existing concepts of self and others. We assume that there are no pre-existing frames of self and other and that these concepts themselves are acquired through learning (Fig. 1c). From the viewpoint of an agent, the concepts of self and other are acquired in one and the same process of experience, starting from a situation where there is neither self nor other.

Obviously, the sensations given to an agent in the first stage do not have any particular indications that they belong to the self or to the other. For the very agent, these sensations are nothing more than external stimuli, even if they are considered sensations belonging to a particular body from the third-person perspective. Using these “anonymous” sensations, we must obtain the representations of self and other as a result of learning. If this assumption holds, what mechanism makes it possible to learn and obtain the representations of self and other starting from a stage in which there is no clear distinction between them? If we can specify the underlying mechanism and reconstruct the process of learning that is enabled by it, our assumption will be more plausible.

**Figure 2:**
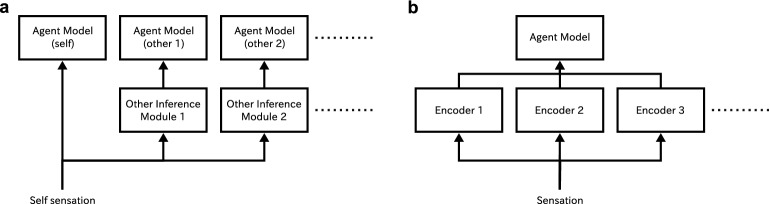
Two different conceptual models of the self and the other in sensory processing. (**a**) Model with multiple internal agent models for the self and others. (**b**) Model with a single internal agent model.

First, let us outline the requirements for a possible mechanism at the conceptual level. In the initial stage, given that self is not clearly distinguished from other, we have no reason to think that given sensory information must be used on behalf of only one agent. There may be a mechanism by which the given sensory information is used in multiple processing paths, which give us multiple ways to interpret the sensory information. This does not mean that socially interacting agents (at least humans) have one circuit for each of the other agents and process the information for each agent separately. This one-to-one correspondence between circuits and agents would be unreasonable because, in this case, the agents would need as many circuits inside them as the number of other agents they encounter. Furthermore, they do not have circuits in advance that correspond to self and other (Fig. 2a). It is more natural to assume that each agent has only one circuit that models any agent (including the self). In this case, the given sensation is processed through multiple paths, and the results are then processed by a single circuit (Fig. 2b). That is, an agent uses the same, single neural circuit for “anyone” in general. We can assume that social agents obtain a single concept of agent while distinguishing self and others at the same time. This assumption allows us to consider that self and others can be equated to each other at a certain level without abolishing their differences.

Based on these ideas, we propose a novel neural mechanism that can build a single internal model that is applicable to “anyone,” which we call the “superposition mechanism.” In this mechanism, a single flow of sensory inputs is encoded by two different neural modules, and the sensory features that are encoded in different ways are then further processed by a single module without distinction. By this process of duplication and superposition, we can model the above-mentioned specific relationship between self and other. The representations of self and other are not given in the mechanism in advance but obtained only by learning.

Of course, it is known that self-other differentiation involves a great variety of interactions between infants and their caregivers or other people. Obviously, it is impossible to simulate all of these interactions. We attempted to determine by simulation what kind of mechanism would, at a minimum, allow us to differentiate between ourselves and others while also relating them. To keep the conditions minimal, we decided to provide only visual and motor information to the agent. Our model cannot simulate all of the diverse and complex processes of self-other differentiation but only abstractly simulates the perspectival representations that depend on the perspectives of self and others. Although it is difficult to map this ability to a specific stage of infant development, there is no doubt that this ability is so fundamental that without it, the understanding of self and others, and thus social cognition, would be impossible.

## Result

Based on our hypothesis, we conducted experiments by implementing the “superposition mechanism” using a deep neural network^17^. In our experiment, an agent with the superposition mechanism moves around in a virtual environment where another agent is present and learns only to predict visual sensation. Then, we analyzed the internal representations developed as the result of the predictive learning to investigate how they can be interpreted in terms of the frames of “self” and “other.”

### Superposition network

**Figure 3:**
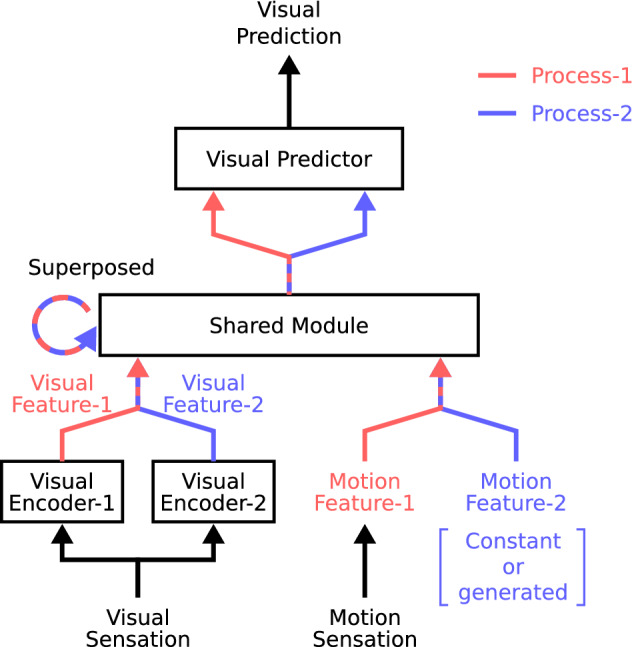
Schematic view of the superposition network. Red and blue lines indicate the processing of process-1 and process-2, respectively. Striped lines with red and blue indicate two parallel processes.

Our superposition mechanism, shown in Fig. 2b, was implemented by a deep neural network model. We call the constructed model the superposition network (Fig. 3). Deep neural networks have recently been used to simulate the development of various cognitive abilities through learning^18–21^. Our proposed superposition network, implemented on a simulated agent, was trained to predict future vision based on the history of visual and motor sensations, and the developed abilities were investigated. In the current study, we consider a situation with two agents, agent-1 and agent-2, where the superposition network implemented on agent-1 learns the visuomotor sensations of agent-1.

The superposition network as a whole is trained to predict the visual sensations of agent-1 by receiving visual and motor sensations of agent-1. We briefly described the processes within the superposition network (see the “Methods” for the details). The input sensations propagate through two different process paths, i.e., process-1 and process-2. First, visual sensations are converted into visual features by two encoders (Visual Encoder-1 and Visual Encoder-2). For the proprioceptive sensations of motion, the raw motion commands of agent-1 are used without any conversions. Then, these two visuomotor features in the two process paths are processed by the same shared module (Shared Module). Shared Module is a recurrent neural network with a long short-term memory (LSTM)^22^, which can retain memories. This means that Shared Module can process the current sensory features based on the memories of past sensory features and processing results. It should be noted that using Shared Module does not mean that all the information is processed in a mixed manner together. The memories are maintained independently for process-1 and process-2 and processed separately so that Shared Module generates two separate outputs. However, the use of Shared Module imposes the constraint that the way of processing must be the same. This allows Shared Module to simultaneously generate two different interpretations of a single sensation using the same generation manner. A subsequent module (Visual Predictor) integrates the two outputs of Shared Module in a mixed manner to predict future visual sensations.

Although modules in the superposition network (Visual Encoder-1 and Visual Encoder-2, Shared Module, and Visual Predictor) have different structures by design, these modules have no specific function at the initial stage, similar to infants, as the neural weight parameters are randomly initialized. The superposition network is trained to predict future vision. The learning process of the model is designed according to the predictive coding/processing theory^23^ (which, incidentally, has attracted attention as a theory that provides an unified explanation of brain functions). It is already known that predictive learning enables deep neural network models to learn abstract representations in a self-supervised manner^24–26^.

### Shared spatial representation

**Figure 4:**
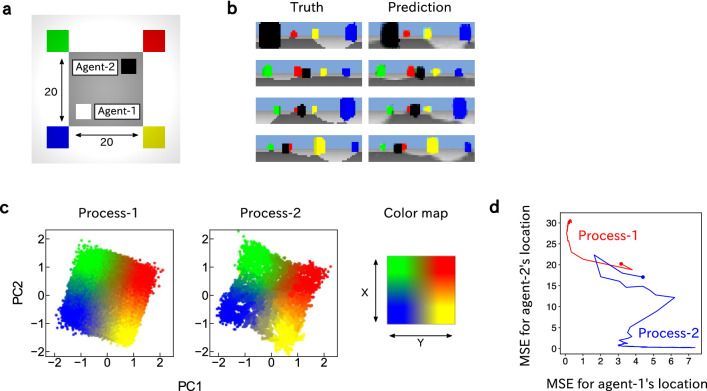
Spatial representation of agent-1 and agent-2 obtained in the superposition network. (**a**) The simulated environment where agent-1 and agent-2 exist. The agents sense a $360^{\circ }$ panoramic view as a vision via the omnidirectional camera. (**b**) Visual images obtained by agent-1. The ground-truth visions (left) and the visions predicted by the trained superposition network (right) are shown. The black object shown in each image is agent-2, and the red, green, blue, and yellow objects are visual landmarks. (**c**) Neural activations of process-1 and process-2 of Shared Module. The neural activations of both processes are displayed separately in two-dimensional space by PCA, with the colors indicating the agent’s location (see “Methods”). Left, process-1’s neural activations, which are colored based on agent-1’s location; middle, process-2’s neural activations, which are colored based on agent-2’s location; right, a color map that defines the mapping from the agent’s location to the color of the neural activations. The variances explained by the first and second principal components were 0.45 and 0.42, respectively. (**d**) Regression analysis on the neural activations of Shared Module for process-1 and process-2. The mean squared error between the true locations of the agents and the locations predicted from the neural activations by linear regression models are shown. Red and blue are the results of regressions from the neural activations of process-1 and process-2 to the locations of agent-1 and agent-2, respectively. The lines show the changes in the regression errors during learning, while the points indicate the beginning of learning.

Our simulated environment is shown in Fig. 4a. The superposition network was trained to predict agent-1’s vision. Within a single trial, agent-1 moves around in the environment. In contrast, agent-2 does not move within a single trial but is instead randomly placed at the beginning of each trial. To correctly predict visual sensation, the network is expected to learn to recognize agent-2’s locations. In this experiment, the motion commands of agent-1 themselves were used as the motion feature of process-1, and a zero vector was used for process-2.

After the training, the superposition network was able to predict future vision, including agent-2’s appearance (Fig. 4b and Supplementary Video 1). We visualized the neural activations of Shared Module in two-dimensional space by using principal component analysis (PCA) (see the “Methods” for details) and found that the neural activation patterns of process-1 and process-2 represented the locations of agent-1 and agent-2, respectively; the neural activation patterns shown in the PCA space are arranged in correspondence with the spatial relationships of the agents’ physical locations. Thus, the superposition network developed the representations of spatial location for both agent-1 and agent-2 simultaneously in the single Shared Module. Notably, the same neural activations were induced in process-1 and process-2 (which correspond to agent-1 and agent-2) for the same physical locations (Fig. 4c and Supplementary Video 1). The neural activation patterns that we found are considered to be similar to the social place cells that represent individuals’ spatial locations without distinguishing the individuals themselves^27–29^. That is, neurons that fire when an agent is in a certain location also fire when it observes that other agents are in the same location. It is as if its own location and the locations of other agents are encoded in the same module. We also observed a gradual differentiation of the representations of spatial locations in Shared Module by regression analysis of the agents’ locations from neural activations (Fig. 4d). In the early stages of learning, the agents’ locations could not be accurately predicted from the neural activities of either process-1 or process-2, but as learning progressed, the locations of agent-1 and agent-2 could be well predicted from the activities of process-1 and process-2, respectively. In other words, the networks gradually developed the representation of the spatial locations of agent-1 and agent-2 starting from no representation of the agents.

### Perspective-taking by decoding

The above analysis of Shared Module revealed that the two different but shared representations of spatial locations of agent-1 and agent-2 are obtained in process-1 and process-2, respectively, through predictive learning. During predictive learning, the visual encoders also learned to encode the same visual sensation as different visual features to organize the spatial representations of agent-1 and agent-2. Then, we investigated what visual features are encoded by decoding them as visual sensations. Visual decoding allows us to identify what the network “sees” in process-1 and process-2.

**Figure 5:**
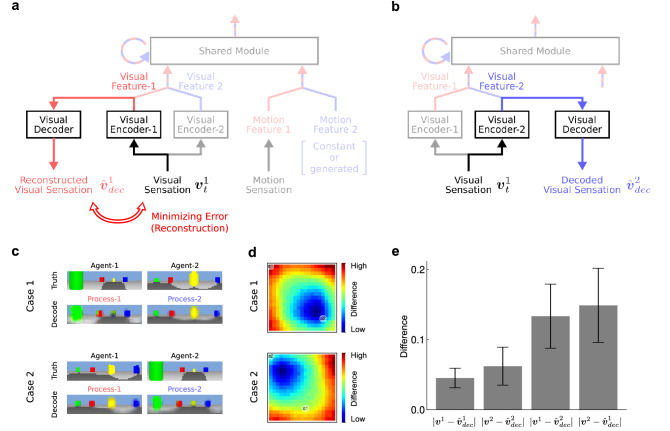
Visual decoding from the parallel-processed visual features. (**a**) Reconstruction of agent-1’s vision from process-1’s visual feature by Visual Decoder. (**b**) Decoding of process-2’s visual features as agent-1’s vision by Visual Decoder. (**c**) Example reconstructed and decoded visions from the visual features of process-1 and process-2. The ground-truth visions of agent-1 and agent-2 (left), and the reconstructed and decoded visions (right). The visions are displayed for two different placements of agent-1 and agent-2. (**d**) Heatmaps showing the differences between the decoded visions and agent-2’s true visions at each location over the arena. The heatmaps are displayed for two cases where the placements of agent-1 and agent-2 were the same as those for each case in (**c**). (**e**) Histogram showing the differences between the reconstructed or decoded visions and the ground-truth visions of agent-1 and agent-2. Error bars indicate the standard deviations of the errors [see “Methods” for the details of (**d**) and (**e**)].

To decode the feature vectors, we first trained an additional visual decoder network (Visual Decoder) to reconstruct the input visual sensation from the encoded feature vectors of process-1 (Fig. 5a). Although it is only trained for process-1, the trained Visual Decoder can also be used to decode the visual features of process-2 (Fig. 5b), as the structure of the visual encoders are the same and the encoded features are used as inputs to Shared Module. By decoding the visual features of process-2, we can visualize them in the form of visual sensation. The decoding process is considered to be the interpretation of what the module sees in process-2 (see “Methods” for details).

Then, we found that the decoded visual sensations were similar to what agent-2 actually sees (Fig. 5c–e, and Supplementary Video 2), which was never provided beforehand to the network. This result means that the superposition network interpreted the single visual sensation to construct not only the view from the perspective of agent-1 but also that from the perspective of agent-2. It also means that the superposition network acquired the basic ability for visual perspective-taking, which is to understand what an agent sees when it stands at another agent’s location.

### Generating another agent’s motion

During predictive learning, agent-2 did not move over time. The superposition network was also trained for the case where agent-2 moved. Agent-2 moved according to three different policies of movement patterns. In situations where agent-2 moves, to correctly predict visual sensation, the network has to take into account the changes in time that are not caused by agent-1’s movements.

**Figure 6:**
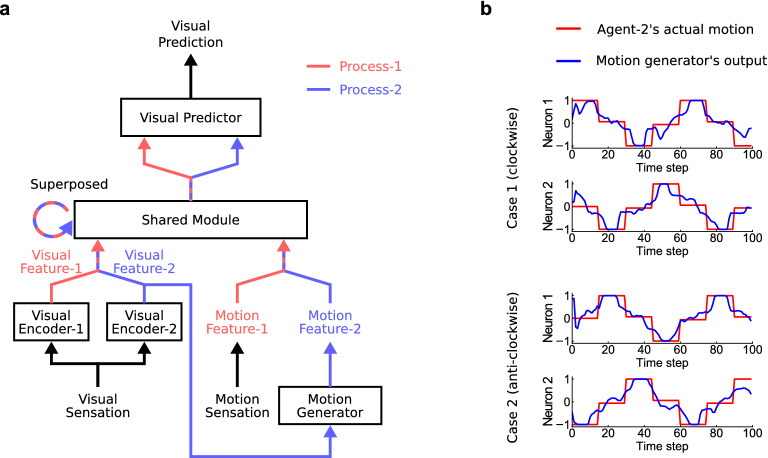
Generation of agent-2’s motion by the superposition network. (**a**) Schematic view of the superposition network with Motion Generator. (**b**) Sequences of Motion Generator’s outputs while Motion Generator was receiving visual features, where agent-2 was moving under the policies of turning around in the clockwise and anticlockwise directions. Sequences of actual motion are also shown. The correlation coefficients, which were calculated while the network ran over 300 sequences, between the actual motion of agent-2 and Motion Generator’s output were 0.87 and 0.88 for neurons one and two, respectively.

To take into account these changing visual sensations, an additional module was added to the trained superposition network to generate motion features (Fig. 6a). We call this additional module Motion Generator. Motion Generator receives the visual feature and outputs vectors of process-2, which are used as the motion feature of process-2. The motion feature generated by Motion Generator can change the internal states of Shared Module in process-2 and consequently the visual prediction. Motion Generator was also trained only to predict the visual sensation as in the previous experiment and was never supervised using any teaching signals, such that the output became the actual motion of agent-2.

After training, the superposition network was able to correctly predict the future vision of agent-1, including the appearances of mobile agent-2. Then, the neural activations of process-2 were analyzed by PCA in the same way as previously described (Supplementary Video 3). The superposition network predicted future vision by updating the neural activation in correspondence with agent-2’s spatial location by using Motion Generator. We also found that Motion Generator’s outputs when observing agent-2’s behaviors showed similar values to those of agent-1’s motion as if agent-1 had moved around in the same way as agent-2 (Fig. 6b). These developed motor representations shared between agent-1 and agent-2 are conceptually similar to mirror neurons in the sense that the observation of agent-2’s behaviors from the perspective of agent-1 elicits mirroring neural activations that occur when agent-1 is performing the same behaviors^30^. The results indicate that predictive learning alone allows the superposition network to interpret visual sensation to construct the motion command that should have generated agent-2’s behavior.

## Discussion

Inside agent-1, we identified the neural activation patterns that correspond to the actual places and visual perspectives of agent-1. These can be interpreted as the representations of the “self” for agent-1. Inside agent-1, we also found the activation patterns that correspond to the actual places, visual perspectives, and motion commands of agent-2. These can be regarded as the representations of the “other” for agent-1. As we reported, the representations found inside agent-1 contain characteristics similar to those of social place cells and mirror neurons. This result strongly suggests that the representations in question correspond to what we call the representations of “self” and “other.” It is important to note here that the frames of “self” and “other” were not given in advance to agent-1. Rather, agent-1 acquired the above representations of “self” and “other” through learning only.

TT and ST seem to assume that there is a pre-given framework for the self and others that is already present when one starts to understand others. According to these theories, agents fill in the blanks corresponding to other people’s minds with certain contents using theories of mind or simulations. However, it is possible to assume that agents begin their experience from undifferentiated general perceptions and acquire the concepts of self and other through learning only (IT does not mention this explicitly). Our experiments showed that this is actually possible. To understand others, agents do not need to have a pre-given framework with empty spaces reserved for the self and other. Instead, they need to have only a minimal mechanism for processing in a shared module information that has been processed through multiple paths. This simple mechanism of duplicating and superposing information is a possible candidate for the minimal requirement for social cognition.

According to TT and ST, an agent needs to “reuse” its own pre-existing internal information to understand others (using theories of mind or simulations). In contrast, the superposition mechanism we propose allows agents to process information about the self and others without distinguishing them from the beginning. As it were, at a certain level, one can understand others as one understands oneself (and vice versa). We can also add that in the superposition mechanism, understandings of others are not achieved by using high-level cognitive functions such as theories of mind or simulation on the basis of a pre-given framework of self and other. Instead, the basis for understanding others develops simultaneously with the development of concepts of self and other.

Our superposition mechanism can be linked to other findings in social cognitive neuroscience. Recent studies have provided evidence that these shared activations for self-experiences and empathic experiences for others are actually shared representations between them rather than just correlated activations^31,32^. The superposition mechanism that enables the acquisition of shared representations of self and other is consistent with these findings. The self-other distinction observed in the superposition mechanism is also related to the real brain. The right temporoparietal junction (rTPJ) is supposed to be responsible for the distinction between self and other in the brain^32–34^, as well as the visual perspective-taking ability^33,35^. In the superposition network, visual encoders perform functions equivalent to visual perspective-taking, as well as the distinction between self and other from a single visual sensation. Therefore, we can assume that the visual encoders in the superposition mechanism play a similar role to the social functions of the rTPJ in the biological brain. The inferior parietal lobule (IPL), which is adjacent to and partly overlaps rTPJ, is also known to be related to visual perspective-taking^36^. This fact enables us to relate the superposition network to further empirical findings. It should be noted that the visual encoders did not acquire self-other distinction and visual perspective-taking as independent functions but rather acquired the self-other distinction in the form of visual perspective-taking. This is consistent with the consideration that visual perspective-taking is necessary for self-other distinction^36,37^. Further, the core of the superposition mechanism, which simultaneously incorporates the seemingly contradictory processes of sharing the module and distinction by two different paths, might be related to the finding that the self-other distinction enhances the abilities of empathy and social cognition^11,38,39^. Finally, although not investigated in the current study, the superposition mechanism possibly explains the confusion between self and other. A study using transcranial magnetic stimulation (TMS) showed that when the function of the right IPL is impaired by TMS, performance in self-other distinction is disrupted^40^. Taking a similar approach, for example, if the visual encoders, which are possibly equivalent to the rTPJ and IPL, are impaired and information about others is passed to the path for self, or vice versa, self-other confusion could occur. This confusion of self and other is a natural consequence of the use of the shared module.

Herein, we describe the limitations of the current simulation study. First, our model does not reflect the complexities of the real world. The environment and agents’ motions are reduced to a simplified form. Recent machine learning and deep learning research have developed more powerful and flexible architectures to deal with complex data^41,42^. It could be possible to extend the superposition network to real world situations by introducing these advanced architectures. However, this would make the model too complex and make it difficult to obtain conceptually clear insights. Our simplified model allowed us to clearly demonstrate the development of “self” and “other” under the superposition mechanism. Of course, we acknowledge that there are still important factors in real-life situations that our model does not adequately reflect. For example, in the actual development of “self” and “other” in humans, there is a non-negligible asymmetry between the amount of experience we have about ourselves and others. In the current simulation, agent-2 is always present in the situation given for agent-1; however, in the real world, there can be situations where no other agents are present and an agent is interacting with the environment alone. This kind of asymmetry definitely affects the developmental process of “self” and “other.” By introducing asymmetries into the simulation, it may be possible to investigate, for example, how an agent’s ability to differentiate itself from others varies depending on the degree of asymmetry, including the presence or absence of others. This is an interesting topic of research, but here we have tried to keep the situation at its simplest, so that only the most basic structures stand out clearly.

Second, the high-level cognitive abilities of mentalizing and mind-reading, i.e., inferring others’ internal mental contents such as thoughts or beliefs and attributing them to the others, were not obtained by the superposition network. What this study investigated was not mentalizing and mind-reading themselves but rather a mechanism that underlies these abilities. Mentalizing and mind-reading presuppose both that the self and other are differentiated and that the other is an agent analogous to the self. This means that mentalizing and mind-reading require a more basic ability that we model in our superposition mechanism. The current model is not expected to recognize false beliefs^43^ that others have about the world (e.g., about the location of objects). There are some studies that model mentalizing or mind-reading, such as the ability to recognize that others have false beliefs^44,45^, but they have only cut out a limited aspect of social cognition as a target. This is a highly developed aspect of social cognition which does not represent a basic ability that can be applied to all other aspects of social cognition. In contrast, we targeted the most basic ability that is prerequisite for social cognition in general, including mentalizing and mind-reading.

Third, whether a mechanism equivalent to the proposed superposition mechanism exists in the biological brain remains an open question. This question is beyond the scope of our paper. However, it may be possible to add some statements regarding this issue. It has been shown that there exist shared neural activations when we empathize with others^46,47^. Certainly, their contribution to social cognition is still controversial, but there are more than a few researchers who support the idea that there is a link between shared neural activations and social cognition^32,48^. It is natural to assume that shared neural activations are strongly related to our experiments. However, the direction of our question differs from that of the majority of the neuroscientists discussing this issue. In neuroscience, the main question is whether the shared neural activations contribute to social cognition, and if so, how they are used for social cognition. However, our theoretical simulation model is intended to show how such shared neural activations can be developed. Our results demonstrated that if the superposition mechanism exists in the brain, the basic abilities to support social cognition can be acquired naturally. This means that the shared neural activations can be interpreted as a consequence of the superposition mechanism. Although this remains a hypothesis, it can be tested by neuroscience in terms of our superposition mechanism.

In conclusion, as suggested above, our results have implications for the neuroscience of social cognition. They shed light on the question of how social place cells, visual perspective-taking, and mirror neurons are formed in human agents. These abilities to understand others are obtained not through supervised learning with engineered teaching data but rather only through sensory experiences such as vision and motion (as occurs in infants). We consider that the abilities achieved in our experiments are just a few examples of the abilities enabled by the superposition mechanism. Our results suggest that various kinds of the abilities to understand others can be explained on the basis of the superposition mechanism, which may provide a new stimulus for studies of social cognition.

## Methods

### Virtual environment

#### Environment and agents

The agents move within a gray, square arena in the simulated environment. The size of the arena is $20 \times 20$, and the center of the arena is defined as the origin, with coordinates (0, 0). Consequently, the four corners of the arena are (10, 10), $(10, -10)$, $(-10, -10)$, and $(-10, 10)$. Four colored cubes are placed as visual landmarks at the corners of the arena. Agent-1 randomly moves within the arena by selecting its destination randomly at one unit distance per environmental time step and remains stationary for periods of time with random intervals. The agents do not change their direction; they are equipped with omniwheels and can move in any direction while sensing an omnidirectional view with an omnidirectional camera. Agent-2 appears as a black cube. For simplicity, collision between agent-1 and agent-2 are not simulated.

##### Visuomotor sensations

At every environmental time step *t*, agent-1 performs motion $m^1_t$ just after it observes vision $v^1_t$. Motion $m^1_t$ is represented as a two-dimensional vector that consists of the x and y displacements of agent-1 in a single time step. The visual image captured by agent-1 $v^1_t$ is of size $16 \times 64$ and consists of three channels (RGB) in each pixel.

### Deep neural networks

We implement our superposition network by using artificial neural networks, particularly deep neural networks^17^. Deep neural networks consist of many layers of artificial neurons. The neurons between or within the layers are connected to each other similar to synapses in a biological brain. The connections have parameters called weights that increase or decrease the signals propagating through the connections. When a network with many layers receives an input, the input propagates through these layers via weighted connections. The weighted inputs received by each neuron and the biases, another set of parameters, are summed, and an activation function is applied to the sum to produce the output. The activation function is usually a nonlinear function such as a logistic sigmoid or ReLU. The entire function of the network, which iteratively transforms the input through weighted sums and nonlinear activation, is initially just random or not actually a valid function. Different architectures present with different connectivities between neurons, such as convolutional neural networks (CNNs) and recurrent neural networks (RNNs), but even in these cases, the weights and biases are initially random, and the networks do not have specific functions from the beginning. The neural network organizes its function by adjusting the weight and bias parameters to achieve some objective. This process of adjustment is called learning. In recent years, deep neural networks have been able to outperform humans in games and tasks following the learning process. In particular, there have been reports of deep neural network models that show behaviors and aspects of neural activities analogous to those of biological brains through learning^18–20^, which have attracted attention as models for the development of cognitive functions^21^.

### Processing of the superposition network

The superposition network consists of a shared module (Shared Module) $\phi _{shared}$, two visual encoder modules $\phi _{enc}^1$ and $\phi _{enc}^2$ for process-1 and process-2, respectively (Visual Encoder-1 and Visual Encoder-2), and a visual predictor module (Visual Predictor). At each time step, the superposition network receives the visual and motor sensations of agent-1 (${v}_{t}^1$ and ${m}_{t}^1$) and generates a visual prediction (${\hat{v}}_{t+1}^1$). The one step process of the superposition network is defined by the following set of equations:

1 $$\begin{aligned} f_{v,t}^1=  \phi _{enc}^1(v_{t}^1), \end{aligned}$$

2 $$\begin{aligned} f_{v,t}^2= \phi _{enc}^2(v_{t}^1), \end{aligned}$$

3 $$\begin{aligned} (h_t^1, c_t^1)= \phi _{shared}(f_{v,t}^1, f_{m,t}^1, h_{t-1}^1, c_{t-1}^1), \end{aligned}$$

4 $$\begin{aligned} (h_t^2, c_t^2)= \phi _{shared}(f_{v,t}^2, f_{m,t}^2, h_{t-1}^2, c_{t-1}^2), \end{aligned}$$

5 $$\begin{aligned} {\hat{v}}_{t+1}^1=  \phi _{pred}(h_t^1, h_t^2). \end{aligned}$$

Equations (1) and (2) denote the encoding of visual sensation by Visual Encoder-1 $\phi _{enc}^1$ and Visual Encoder-2 $\phi _{enc}^2$, respectively. Visual Encoder-1 and Visual Encoder-2 are CNNs. CNNs possess local connectivity between neurons in the hierarchy of layers inspired by the visual cortex of the biological brain and can effectively extract and integrate features such as edges or shapes in visual images by the operation called convolution. Through processing by the visual encoders, a single visual image $v_t^1$ from agent-1’s visual sensation is transformed into compact feature vectors $f_{v,t}^1$ and $f_{v,t}^2$. The two visual encoders have the same structure (see the Supplementary Methods for details) but different weight and biases, and thus the two transformed visual features $f_{v,t}^1$ and $f_{v,t}^2$ are different even though the same visual sensation is input to the visual encoders.

Equations (3) and (4) denote the processing by Shared Module $\phi _{shared}$ for process-1 and process-2. Shared Module is a recurrent neural network. Recurrent neural networks possess recurrent connections of neural weights and can hold information over time through these recurrent connections. We use a long short-term memory (LSTM)^22^ network, a special type of recurrent neural networks, for Shared Module in the superposition network. The neural activations passed through recurrent connections are often called internal states. LSTM has two types of internal states called hidden and cell states, $h_t$ and $c_t$, respectively. In this paper, we only call the hidden states $h_t$ the internal states. The same Shared Module is used for both process-1 and process-2. It means that the visuomotor features of process-1 ($f_{v,t}^1$ and $f_{m,t}^1$) and process-2 ($f_{v,t}^2$ and $f_{m,t}^2$) are processed in the same way following our proposed superposition mechanism. By processing the visuomotor features through two processes, two internal states for both processes, $h_t^1$ and $h_t^2$, are output by Shared Module. The motion feature of process-1, $f_{m,t}^1$, is agent-1’s motion itself, $m_t^1$. The motion feature of process-2, $f_{m,t}^2$, is a zero vector in the experiment where agent-2 does not move and is generated by Motion Generator in the experiment where agent-2 moves.

Equation (5) denotes the generation of visual prediction by Visual Predictor $\phi _{pred}$. The two vectors of the internal states of process-1 and process-2, $h_t^1$ and $h_t^2$, are first concatenated as a single vector and further processed by Visual Predictor to generate a visual prediction. Visual Predictor is also a CNN. In contrast to the visual encoders, through processing by Visual Predictor, the compact feature vectors are transformed into visual images.

### Predictive learning experiment

#### Predictive learning

The parameters of the superposition network are randomly initialized, and initially, the network does not have any specific function. The parameters of the superposition network are optimized based on the gradient with respect to the prediction error for visual sensation, which is often called loss, as the target of minimization. To perform gradient based optimization, the errors between the predicted visual sensation and true future visual sensation (that is, loss) are calculated as follows:

6 $$\begin{aligned} {\mathscr {L}}_{pred} = \sum _{t=1}^T \left\{ \lambda _{{\mathrm{{MSE}}}} {\text{MSE}}({\hat{v}}^1_{t+1}, v^1_{t+1}) + \lambda _{{\mathrm{{MAE}}}} {\text{MAE}}({\hat{v}}^1_{t+1}, v^1_{t+1}) \right\} , \end{aligned}$$

where *T* is the duration of a single sequence ($T=100$), ${\mathrm {MSE}}$ and ${\mathrm {MAE}}$ are the mean squared error and mean absolute error loss functions, respectively, between two vectors, and $\lambda _{{\mathrm{{MSE}}}}$ and $\lambda _{{\mathrm{{MAE}}}}$ are the coefficients for controlling the importance of these loss terms. ${\mathrm {MSE}}$ and ${\mathrm {MAE}}$ are defined as follows:

7 $$\begin{aligned} {\mathrm{{MSE}}}({\hat{y}}, y)= \frac{1}{N}\sum _{i=1}^N ({\hat{y}}_{i} - y_{i})^2, \end{aligned}$$

8 $$\begin{aligned} {\mathrm{{MAE}}}({\hat{y}}, y)= \frac{1}{N}\sum _{i=1}^N |{\hat{y}}_{i} - y_{i}|, \end{aligned}$$

where *N* is the number of elements in each vector for which the error is calculated. By minimizing the loss defined in Eq. (6), the parameters of the network are optimized for predicting visual sensations. The ${\mathrm {MSE}}$ loss produces a larger gradient for a larger error and a smaller gradient for a smaller error. On the other hand, the ${\mathrm {MAE}}$ loss penalizes errors equally regardless of their extent. This means that the ${\mathrm {MAE}}$ loss produces a larger gradient for a small error than the ${\mathrm {MSE}}$ loss. By changing the coefficients for these losses, we can control how much the network learns to minimize the small errors. Additionally, weight decay, which penalizes larger values of the parameters, is applied to all network parameters except for the biases. By introducing weight decay, overfitting to the training data can be mitigated.

To calculate the gradient of the parameters, we use backpropagation through time (BPTT)^49^. To update the parameters with the calculated gradients, the stochastic gradient descent method is used. Specifically, we use the Adam algorithm^50^. The superposition network is trained 400 times over the training sequences with a mini-batch size of 10. During the first 200 iterations of training, $\lambda _{{\mathrm{{MSE}}}}=1$ and $\lambda _{{\mathrm{{MAE}}}}=0$, while during the last 200 iterations of training, $\lambda _{{\mathrm{{MSE}}}}=0$ and $\lambda _{{\mathrm{{MAE}}}}=1$. In the last 200 iterations of training, all parameters except for those of Visual Predictor are fixed. In other words, only Visual Predictor is fine-tuned to reduce small errors. Thus, the later training has an effect on the sharpness of the predicted image, but it does not affect how the internal state of Shared Module is organized.

##### Visual input masking

For the development of the internal model of the external spatial structure, the visual input to Shared Module is masked randomly. Concretely, the encoded visual feature vectors $f_v^1$ and $f_v^2$ from the two visual encoders are replaced with zero vectors with 99% probability at each time step. This masking makes the LSTM learn to update its internal states by integrating the motion input sequences for correctly predicting visual images.

##### Training sequences

For the prediction training, we collect 1000 of agent-1’s visuomotor experiences with a duration of 100 time steps during which agent-1 moves around while agent-2 does not. At the beginning of each sequence, the positions of agent-1 and agent-2 are initialized randomly.

#### Structure of shared module

The LSTM constituting Shared Module has 128 hidden units. The superposition network has two copies of the LSTM module as Shared Module $\phi _{shared}$. Each of the two copied LSTMs has its own internal states $h^1$ and $h^2$, which are updated by receiving visual and motion feature inputs, respectively. The two LSTMs receive visuomotor inputs by the same receptor neurons, and these inputs are processed in the same way using the same network weights and biases. During the training using backpropagation, as described in the following section, gradients are propagated to the two LSTMs through two separate processing paths. The gradients of both LSTM copies are summed and used to update the parameters of the LSTMs. In this sense, the LSTM modules in process-1 and process-2 are shared.

#### Structure of visual encoder and visual predictor

Visual Encoder-1 $\phi _{enc}^1$ and Visual Encoder-2 $\phi _{enc}^2$ both have the same structure, which consists of three convolutional layers followed by a fully connected layer; however, these visual encoders have different weights and biases (see the Supplementary Methods for details of the visual encoders’ structures). To ensure that the visual feature vectors $f_v^1$ and $f_v^2$ do not have large differences in their values, layer normalization^51^ is used for the fully connected layer of both visual encoders. Visual Predictor $\phi _{pred}$ consists of three transposed convolutional layers following a fully connected layer (see the Supplementary Methods for details). The fully connected layer of $\phi _{pred}$ takes the two parallel internal states of Shared Module as inputs and integrates them into a single-feature vector.

### Visual decoding experiment

#### Reconstruction learning with agent-1’s visual sensation

To visualize the visual features encoded by the visual encoders, the visual decoder (Visual Decoder) $\phi _{dec}$ is trained to reconstruct the visual sensation of agent-1. Please note that we use a single Visual Decoder, whereas two different visual encoders are used. The visual features encoded by Visual Encoder-1, as depicted in Eq. (1), are input to Visual Decoder, and the visual images are generated as the reconstruction of the input visual sensation.

9 $$\begin{aligned} {\hat{v}}_{dec}^1 = \phi _{dec}(f_v^1). \end{aligned}$$

During the training of Visual Decoder, only the visual features from Visual Encoder-1 are used as input; those from Visual Encoder-2 are never used.

For optimizing Visual Decoder, the following reconstruction error is calculated as the loss:

10 $$\begin{aligned} {\mathscr {L}}^{rec} = {\mathrm{{MAE}}}({\hat{v}}^1_{dec}, v^1) \end{aligned}$$

The gradients of the loss ${\mathscr {L}}^{rec}$ are calculated simply by the backpropagation algorithm. By using the calculated gradient, the parameters of Visual Decoder are updated by the Adam algorithm in the same manner to the above predictive learning. This reconstruction training of Visual Decoder is similar to that of an auto-encoder^52^, in which an encoder that encodes the input and a decoder that decodes the encoded feature vectors are trained simultaneously; however, in our experiment, the visual encoders have already been trained through predictive learning and are fixed during the reconstruction training, and only Visual Decoder is trained. Visual Decoder is trained 10 times over the training data with a mini-batch size of 10. Weight decay is also applied to all of Visual Decoder’s parameters except for the biases.

##### Training visual sensations

The visual images collected for the above predictive learning, that is, agent-1’s visual sensation, are also used for the reconstruction learning for Visual Decoder.

#### Decoding agent-2’s visual sensation

After the reconstruction training, the visual features from Visual Encoder-2 are decoded by using the trained Visual Decoder. To decode the visual features from Visual Encoder-2 after the training, the visual features from Visual Encoder-2 can be simply input to Visual Decoder, as Visual Encoder-2 has the same structure as Visual Encoder-1;

11 $$\begin{aligned} {\hat{v}}_{dec}^2 = \phi _{dec}(f_v^2). \end{aligned}$$

The decoded vision is evaluated in terms of the similarity between the visual sensations of agent-1 and agent-2.

#### Evaluation of the decoded vision

To evaluate the decoded vision in the visual decoding experiment, we collect visual images for uniformly distributed agent locations as follows. In this case, agent-1 and agent-2 are placed at locations whose x- and y-coordinates are integers within the range $[-9, 9]$. The visual images are obtained for all possible combinations of placements of agent-1 and agent-2. For each placement of agent-1 and agent-2, the decoded visual image is obtained from agent-1’s visual sensation, and the differences between the decoded vision and actual agent’s vision are evaluated. To display how the visions decoded from the visual features from Visual Encoder-2 depend on the actual location of agent-2, the differences between the decoded vision and the visions of agent-2 for each location over the arena are calculated (Fig. 5d). The differences between the decoded vision and actual vision are calculated as absolute differences and averaged over pixels in the visual image. Concretely, the differences between the reconstructed/decoded visions ${\hat{v}}_{dec}^1$/${\hat{v}}_{dec}^2$ and the actual visions of agent-1/agent-2 $v^1$/$v^2$ are calculated, and the calculated differences between the actual vision and reconstructed/decoded vision are averaged over all placements of agent-1 and agent-2 (Fig. 5e).

#### Structure of visual decoder

Visual Decoder $\phi _{dec}$ has the same structure as Visual Predictor and forms an auto-encoder with Visual Encoder-1 $\phi _{enc}^1$. Because Visual Encoder-1 $\phi _{enc}^1$ and Visual Encoder-2 $\phi _{enc}^2$ have the same structure, Visual Decoder could also receive the encoded visual feature vector from Visual Encoder-2 $\phi _{enc}^2$.

### Experiments related to generating another agent’s motion

#### Agent-2’s behavior

To learn when agent-2 is moving, agent-2’s behaviors are controlled by simple deterministic policies: turning within the arena to draw a square in both the clockwise and anticlockwise directions and staying only at the initial location. For the policy of turning and drawing a square, agent-2’s behavior is controlled by periodically determining its destination from points (8, 8), $(8, -8)$, $(-8, -8)$, and $(-8, 8)$. The initial location of agent-2 under this policy is randomly selected from these destination points.

#### Motion generator

To predict when agent-2 is moving, Motion Generator $\phi _{gen}$, which consists of an LSTM and a fully connected layer, is introduced. Motion Generator receives the visual feature of process-2 and outputs vectors of the same size as the motor sensation.

12 $$\begin{aligned} f_{m,t}^2= \phi _{gen}(f_{v,t}^2)). \end{aligned}$$

Please note that Motion Generator uses an LSTM, although it is not denoted explicitly in Eq. (12). LSTMs can take into account the context of the past inputs to generate outputs by using recurrent connections. Thus, with its LSTM, Motion Generator can predict agent-2’s future movements by considering the past visual inputs. The generated motion features are input to Shared Module as in Eq. (4), and the visual predictions are generated following Eqs. (1)–(5).

#### Predictive learning with motion generator

The superposition network with Motion Generator is also trained to predict agent-1’s visual sensations. The prediction loss used to train Motion Generator is the same as that given by Eq. (6); however, only the ${\mathrm{{MAE}}}$ loss is considered ($\lambda _{{\mathrm{{MSE}}}}=0$ and $\lambda _{{\mathrm{{MAE}}}}=1$). To minimize the prediction loss, Motion Generator’s parameters should generate $f_{m,t}^2$ so that it properly updates Shared Module’s internal states to generate the correct visual sensation.

In addition to the prediction of visual images, the superposition network is trained to predict the encoded visual feature vectors $f^1_{v,t}$ and $f^2_{v,t}$; the additional visual feature prediction module $\phi _f$ predicts visual features from the internal states of Shared Module: ${\hat{f}}^1_{t+1} = \phi _f(h^1_t)$ and ${\hat{f}}^2_{t+1} = \phi _f(h^2_t)$, where ${\hat{f}}^i_{t+1}$ is the vector of predicted visual features. The feature prediction module $\phi _{f}$ consists of two fully connected layers with 128 and 64 hidden units. Each fully connected layer is followed by ReLU and layer normalization. The training loss for this visual feature prediction is described as follows:

13 $$\begin{aligned} {\mathscr {L}}_{feature} = \sum _{t=1}^T \left\{ {\mathrm{{MSE}}}({\hat{f}}^1_{t+1}, f^1_{t+1}) + {\mathrm{{MSE}}}({\hat{f}}^2_{t+1}, f^2_{t+1}) \right\} . \end{aligned}$$

This auxiliary loss based on abstract-level features helps the network to generate sharp images in the image generation models^53^. For the case where only image-level prediction loss is used, the prediction error for small objects in images has a small effect on the learning; however, when using an abstract-level feature loss, the size of the objects in the original images is irrelevant. In our case, when agent-2 is distant from agent-1, agent-2 appears small in the image, and we find that this feature prediction loss accelerated the training of Motion Generator. The feature prediction module is trained before Motion Generator, during the training when agent-2 does not move. During the training of Motion Generator, the parameters of the feature prediction module are fixed.

Motion Generator is trained to minimize loss ${\mathscr {L}}_{gen} = {\mathscr {L}}_{pred} + {\mathscr {L}}_{feature}$. BPTT is performed through the sequence, and the gradients of loss are calculated. Then, the parameters of the network are updated. In this experiment, only Motion Generator’s parameters are learned, and the parameters of Shared Module $\phi _{shared}$, Visual Encoder-1 $\phi _{enc}^1$, Visual Encoder-2 $\phi _{enc}^2$, and Visual Predictor $\phi _{pred}$ are fixed. To encourage Motion Generator to generate a proper motion feature for correct visual prediction, the visual inputs for Shared Module are always masked, except at the first time step, and only Motion Generator receives visual inputs. Motion Generator is trained 200 times over the training sequences with a mini-batch size of 10. Weight decay is also applied to all of Motion Generator’s parameters except for the biases.

##### Training sequences

We collect 1000 of agent-1’s visuomotor experiences with a duration of 100 time steps for each of the three policies for agent-2 for a total of 3000 collected sequences. While agent-2 moves following its policies, agent-1 also moves as previously described.

#### Structure of motion generator

Motion Generator $\phi _{gen}$ consists of an LSTM and a fully connected layer. The LSTM of Motion Generator has 128 hidden units. The fully connected layer is followed by a hyperbolic tangent non-linearity. The output motion feature $f_{m,t}^2$ is a two-dimensional vector, the same as agent-1’s motion vector. Through the hyperbolic tangent function, the range of each element $f_{m,t}^2$ is $(-1, 1)$, the same as agent-1’s motion.

### Visualization of neural activation in shared module

To visualize the neural activations in Shared Module, that is, the internal states $h^1_t$ and $h^2_t$, the dimensionality of the internal states was reduced to two by using PCA (principal component analysis). The internal states of process-1 during the processing of all training visuomotor sequences by the trained superposition network were collected. Then, the eigenvectors of the covariance matrix over the collected states were calculated, and the first and second principal components were obtained by projecting the internal states using the first and second eigenvectors. The first and second components of the internal states of process-1 were displayed in two-dimensional space. The internal states of process-2 were mapped onto two-dimensional space by using the first and second eigenvectors obtained from the internal states of process-1; that is, the internal states of process-1 and process-2 were displayed on the same space. The internal states were colored according to the agent’s location. For coloring the internal states, RGB values were assigned to each spatial location; red, blue, green, and yellow, corresponding to the four corners of the arena, and linearly interpolated colors were assigned to the inside of the arena.

## Supplementary Information

Supplementary Information.

Supplementary Video 1.

Supplementary Video 2.

Supplementary Video 3.

## Data Availability

The codes for all of the simulation experiments and analyses in this study are available at https://github.com/wtrnoguchi/superposition.
