# Supplementary material for: Superposition mechanism as a neural basis for understanding others
Source: Sci Rep. 2022 Feb 21;12:2859. doi: 10.1038/s41598-022-06717-3 (PMC8861173; doi:10.1038/s41598-022-06717-3)
Supplement: Supplementary file 1 — Supplementary Information. [file 41598_2022_6717_MOESM1_ESM.pdf]

Supplementary Information for "Superposition mechanism as a neural basis for understanding others"

Wataru Noguchi[1,*], **Hiroyuki Iizuka**[1,2], **Masahito Yamamoto**[1,2], **and Shigeru Taguchi**[2,3]

[1]Faculty of Information Science and Technology, Hokkaido University, Sapporo, 060-0814, Japan

[2]Center for Human Nature, Artificial Intelligence and Neuroscience, Hokkaido University, Sapporo, 060-0812, Japan

[3]Faculty of Humanities and Human Sciences, Hokkaido University, Sapporo, 060-0810, Japan

[*]w.noguchi@ist.hokudai.ac.jp

Supplementary Methods

Details of the visual encoders, decoder, and predictor

The visual encoders (Visual Encoder-1 and Visual Encoder-2), Visual Decoder, and Visual Predictor are CNNs. The visual encoders consist of three convolutional layers and a fully connected layer. The first convolutional layer has 16 filters of size 3×10 with a stride of 2 and padding of 1. The second convolutional layer has 32 filters of size 3×10 with a stride of 2 and padding of 1. The last convolutional layer has 64 filters of size 3×10 with a stride of 2 and padding of 1. The last convolutional layer is followed by a fully connected layer with 64 hidden units. All convolutional and fully connected layers are followed by a rectified nonlinear unit (ReLU). Layer normalization is applied after the fully connected layer and before the ReLU. The scaling and shifting parameters of the layer normalization are shared between both encoders. The parameters of the layer normalization are also optimized during training. Visual Encoder-1 and Visual Encoder-2 have the same structure described above.

Visual Predictor consists of two fully connected layers and three transposed convolutional layers. The first fully connected layer, which has 64 hidden units, integrates two parallel outputs of the superposition module. The second fully connected layer has 256 hidden units, and it is followed by transposed convolutional layers. The first transposed convolutional layer has 32 filters of size 4×10 with a stride of 2 and padding of 1. The second transposed convolutional layer has 16 filters of size 4×12 with a stride of 2 and padding of 1. The last transposed convolutional layer has 3 filters of size 4×12 with a stride of 2 and padding of 1. These transposed convolutional and fully connected layers, except for the last transposed convolutional layer, are followed by a ReLU. The last transposed convolutional layer is followed by a hyperbolic tangent non-linearity. Visual Decoder has the same structure as Visual Predictor except that it does not have the first fully connected layer for integrating the superposition module's outputs.

Captions of Supplementary Videos

Supplementary Video 1. Sequences of the predicted vision and the internal states of the superposition network. An overview of the environment and the ground truth of the vision is shown. The current internal states, which are shown as white points, are plotted over the colored visualization of the internal states, which is the same as in Fig. 4 (c), as the background. Agent-2 does not move, as during the training. The sequences are shown for two selected trials with different placements of agent-2.

Supplementary Video 2. Vision decoded by Visual Decoder from the encoded visual features of process-1 and process-2 ($\hat{v}^1_{dec}$ and $\hat{v}^2_{dec}$). The ground-truth visions of agent-1 and agent-2 (v^1 and v^2) are also shown. The decoded visions are shown as sequences that correspond to the sequence of agent-1's vision while agent-1 is stationary and agent-2 is moving. The video shows how the decoded vision changes depend on agent-2's location. The sequences are shown for three trials with different placements of agent-2.

Supplementary Video 3. Sequences of the predicted vision and the internal states of the superposition network with Motion Generator as in Supplementary Video 1. Agent-1 does not move in this case but did during the training. The sequences are shown for two selected trials with different placements of agent-1 for each policy of agent-2.
